# Mode of delivery among preterm twins and offspring health, a retrospective cohort study

**DOI:** 10.1007/s00431-025-06060-5

**Published:** 2025-03-10

**Authors:** Itamar Ben Shitrit, Eyal Sheiner, Gali Pariente, Ruslan Sergienko, Tamar Wainstock

**Affiliations:** 1 Department of Epidemiology, Biostatistics and Community Health Sciences, Ben-Gurion University of the Negev, Beer-Sheva, Israel; 2 Department of Obstetrics and Gynecology, Soroka University Medical Center, Ben-Gurion University of the Negev, Beer-Sheva, Israel; 3 Department of Health Policy and Management, Ben-Gurion University of the Negev, Beer-Sheva, Israel; 4 Clinical Research Center, Soroka University Medical Center, PO Box 151, 84101 Be’er-Sheva, Israel; 5 Faculty of Health Sciences, Ben-Gurion University of the Negev, Beer-Sheva, Israel; 6 Emergency Pediatrics Department, Faculty of Health Sciences, Soroka University Medical Center, Ben-Gurion University of the Negev, Beer-Sheva, Israel

**Keywords:** Preterm twins, Mode of delivery, Long-term morbidity

## Abstract

Although cesarean delivery (CD) has been linked to long-term health risks in singleton infants, the impact of delivery mode on long-term health outcomes in preterm twins remains underexplored. A retrospective cohort study was conducted at a tertiary medical center in Israel from 1991 to 2021, comparing preterm twins vaginally delivered (VD) versus cesarean section, excluding cases with congenital malformations or perinatal deaths. Kaplan–Meier survival curves were used to compare the cumulative incidence, and Cox proportional hazards models were applied to adjust for potential confounders. Four thousand twenty-eight preterm twin offspring were included, with 1703 (42%) VD and 2325 (58%) by CD. Preterm twins delivered by CD had a higher incidence of respiratory morbidities (42% vs. 35% in the VD group, *p* < 0.001), with an adjusted Hazard Ratio (aHR) of 1.15 (95%CI 1.02–1.30). CD was associated with an increased incidence of neurologic morbidities (22% vs. 17% in the VD group, *p* < 0.001), with an aHR of 1.16 (95%CI 1.02–1.36). CD was associated with a higher incidence of infectious morbidities (69% vs. 62%, *p* < 0.001), with an aHR of 1.10 (95%CI 1.01–1.21). Gastrointestinal morbidities were more pronounced in the CD group (29% vs. 25%, *p* < 0.001), but the multivariable analysis did not reach significance (aHR = 1.10, 95%CI 0.95–1.27). Sub-analyses of elective-uncomplicated deliveries showed consistent results for most morbidities. *Conclusions:* Cesarean delivery in preterm twins is associated with long-term respiratory, neurologic, infectious and gastrointestinal morbidities of the offspring. The findings suggest the potential benefits of vaginal over cesarean deliveries regarding offspring long-term health complications.

**Table:** 

What is Known: • Studies on singleton births show that cesarean delivery may increase respiratory, infectious, neurological and gastrointestinal outcomes remains inconsistent across term and preterm deliveries. • Cesarean delivery rates remain high despite recommendations to reduce their frequency, yet data on the association between cesarean delivery and morbidity among twins, particularly in small for gestational age twins, is limited.
What is New: • This is the first large-scale study demonstrating that cesarean delivery in preterm twins increases the odds of respiratory, neurologic, infectious and gastrointestinal long-term morbidities up to age 18. • The higher rates of respiratory, neurologic, infectious, and gastrointestinal complications persist even in uncomplicated cesarean deliveries.

**Supplementary Information:**

The online version contains supplementary material available at 10.1007/s00431-025-06060-5.

## Introduction

Preterm delivery (PTD), defined as delivery before 37 gestational weeks, is a major public health concern. PTD occurs in approximately 11% of births worldwide [1], with over 10% in the USA in 2020 [2], and 8.7% in Europe in 2014 [3]. PTD is the leading cause of perinatal death and contributes to over 75% of neonatal deaths. It is also the second leading cause of death in children under the age of five years [4, 5]. Moreover, the negative consequences of PTD persist and impact health and well-being in adulthood [1, 6].

Cesarean delivery (CD) has been associated with offspring long-term morbidity. CD-born offspring are at increased risk for health complications [1, 7–10], including a higher incidence of obesity, asthma, and autism spectrum disorder (ASD)[10–12]. However, most studies were based exclusively on singleton deliveries.

According to the Centers for Disease Control, the twin rate in 2020 was 31.1/1000 births in the USA, of which approximately 60% were born preterm and approximately 20% before 34 gestational weeks [2]. Twin pregnancies are associated with a higher incidence of neonatal morbidity and mortality compared to singleton pregnancies, mainly due to the increased likelihood of PTD [13–15].

One of the debates surrounding the management of preterm twin gestations is the optimal mode of delivery, with the ongoing disagreement regarding whether a planned CD is safer than vaginal delivery (VD) [16–24]. The Twin Birth Study, a randomized trial, examined the optimal delivery method for twins by comparing short-term perinatal outcomes between planned VD and CD from 32 + 0 gestational weeks, presenting no immediate fetal or neonatal benefits of CD over VD [21, 24]. Additionally, no difference in adverse neurodevelopmental outcomes was noted in children born via planned CD and those via planned VD up to two years of age [20]. However, the study’s scope was limited, focusing only on infants born after 32 weeks and primarily assessing short-term outcomes besides the neurodevelopmental aspects by the age of two years [20, 21].

This study’s objective was to investigate the association between the mode of delivery in preterm twins and long-term pediatric respiratory, neurologic, infectious, and GI morbidities up to the age of 18. Recognizing the differences in intrauterine dynamics and perinatal risks between twins and singletons, we hypothesized that patterns observed in singletons regarding higher morbidity rates may not directly apply to preterm twins. Therefore, dedicated research is needed to better understand these associations in the unique context of twin gestations.

## Materials and methods

In this retrospective cohort study, all twin deliveries at the Soroka University Medical Center (SUMC) between January 1991 and January 2021 were included. SUMC is an 1100-bed university-affiliated tertiary referral center in Southern Israel and the sole tertiary hospital serving a population of approximately 1.1 million citizens. It is the largest birth center in the country, with more than 17,000 deliveries annually (2021). The exclusion criteria encompassed pregnancies in which either twin had chromosomal aberrations or genetic disorders and perinatal mortality cases [22].

The primary exposure mode of delivery was categorized as CD or VD. Primary outcomes were the first offspring’s hospital admission or community-based diagnoses of respiratory, neurologic, infectious, or GI morbidity, defined as an “Event”, identified using specific ICD-9 codes (see Supplementary Table). Offspring follow-up ceased at the first event or upon censoring, which occurred at age 18, death, or study end (January 2021). Mortality was defined as death for any reason. Gestational age was stratified into groups suggested by the WHO: extremely, very, and moderate to late preterm (24 + 0 to 27 + 6, 28 + 0 to 31 + 6, and 32 + 0–36 + 6 respectively) [25]. Insufficient prenatal care was categorized as having four or fewer prenatal visits or beginning such care in the third trimester. Elective CD included cases of CD due to previous cesarean deliveries, malpresentation, and maternal request. Cases of an urgent CD were due to non-reassuring fetal status, non-progressive labor, cord prolapse, failed instrumental delivery, placental abruption, premature rupture of membranes (PROM), and failed induction.

Data was collected from an electronic database, encompassing demographic, obstetrical, and general maternal and fetal information. This process involved merging three databases: the hospital’s computerized hospitalization database (“Demog-ICD9”), which includes demographic details and ICD-9 codes for diagnoses made during hospitalizations; the hospital’s obstetrics and gynecology department’s computerized perinatal database, containing information recorded post-delivery; and the pediatric outpatient database for outpatient diagnoses (Supplementary eTable diagnoses codes). All records and information were anonymized and de-identified before analysis.

### Statistical analysis

Summary statistics were calculated to describe the sample characteristics. Shapiro–Wilk and Kolmogorov–Smirnov tests were performed to assess the distribution of variables. Skewed distributions were presented as median (IQR), while normally distributed variables were presented as mean (SD). The Chi-square, *t*-test, and the Wilcoxon rank sum tests were used to compare perinatal, obstetrics, and maternal characteristics by mode of delivery. The follow-up period was defined as the time from birth to the first event within each disease domain. Association between mode of delivery and later respiratory, neurologic, infectious, or GI morbidity-related hospitalization of the offspring was performed using a Kaplan–Meier survival analysis, comparing the CD group with the VD group using the log-rank test. The proportional hazards assumption was visually assessed using Kaplan–Meier curves, confirming proportionality risks remained consistent during the follow-up period. Multivariate Cox proportional hazard models, adjusted for clustering within pregnancies using Robust Standard Errors (RSE) [26], were employed to study the association between delivery mode and long-term offspring morbidities while also accounting for maternal recurrence during the study period. To account for potential time-dependent confounding related to changes in clinical guidelines and practices over the 30-year study period, all models were adjusted for the year of birth as a covariate. Variables with differences in univariate analyses (*p* < 0.10) were selected for the multivariable model across each disease domain. Model optimization utilized the lowest Bayesian Information Criterion (BIC) and ANOVA for significant enhancements. The analysis was further stratified by gestational age and type of CD (elective or urgent). A two-sided *p*-value of < 0.05 was considered significant. Statistical analysis was performed using R Studio 4.3.1.

## Results

**Fig. 1:**
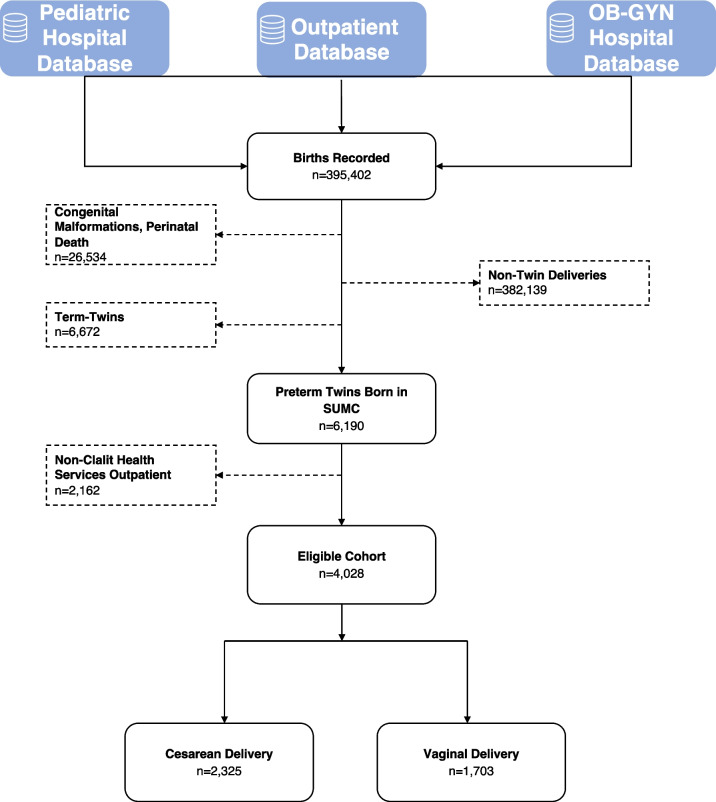
Flow chart

During the study, 395,402 births were recorded, including 382,139 singletons. After excluding 26,534 cases of congenital malformations or perinatal deaths, 13,236 twin births remained, of which 6190 were preterm. Due to missing outpatient data from 2162 offspring covered in other HMOs, 4028 offspring (2014 pregnancies) met the inclusion criteria (Fig. 1). Among these pregnancies, 816 (41%) resulted in both fetuses being delivered vaginally, 1127 (56%) by cesarean, and 71 (3%) had a mixed scenario delivery, with one twin vaginally and the other by cesarean. Individually, 1703 (42%) twins were delivered vaginally, while 2325 (58%) were delivered by cesarean. Table 1 details the populations’ demographic, obstetric, and perinatal characteristics. Compared to mothers with vaginal or mixed deliveries, those who underwent CD were typically older, more likely to conceive following in vitro fertilization (IVF), and to be diagnosed with pregnancy complications, including anemia, gestational diabetes mellitus (GDM), preeclampsia, and placental abruption. No differences were observed between the mode of delivery regarding other characteristics, including different-sex twins, preterm premature rupture of the membranes, and chronic hypertension. Breech presentation was more common in the CD group across all gestational ages, with a consistent incidence of about 50% in each age category (Table 2, S1).

**Table 1:** Baseline maternal and pregnancy characteristics by mode of deliver

Variable	Vaginal, N = 816 (41%)^*1*^	Cesarean, N = 1127 (56%)^*1*^	Mixed, N = 71 (3%)^*1*^
Maternal Age**,** Mean (SD)	28 (5)	30 (6)	29 (6)
Maternal Age Over 35, n (%)	89 (11%)	200 (18%)	13 (18%)
Ethnicity, n (%)			
Bedouin-Arab	485 (59%)	574 (51%)	40 (56%)
Jewish	331 (41%)	553 (49%)	31 (44%)
Gravidity Group*, n (%)			
1	195 (24%)	302 (27%)	16 (23%)
2–4	407 (50%)	517 (46%)	28 (39%)
> 5	214 (26%)	307 (27%)	27 (38%)
Smoking, *n* (%)	1 (0.1%)	9 (0.8%)	0 (0%)
Obesity, *n* (%)	12 (1.5%)	31 (2.8%)	0 (0%)
Anemia, *n* (%)	390 (48.5%)	629 (56%)	43 (61%)
Gestational Diabetes, *n* (%)	43 (5.3%)	89 (7.9%)	7 (9.9%)
Chronic Hypertension, *n* (%)	11 (1.3%)	16 (1.4%)	1 (1.4%)
Maternal History Of Perinatal Death, *n* (%)	28 (3.4%)	67 (5.9%)	2 (2.8%)
In Vitro Fertilization, *n* (%)	111 (14%)	286 (25%)	13 (18%)
Insufficient Prenatal Care, *n* (%)	54 (6.6%)	48 (4.3%)	5 (7%)
Premature Rupture of Membranes > 24 h, *n* (%)	10 (1.2%)	20 (1.8%)	1 (1.4%)
Placental Abruption, *n* (%)	3 (0.4%)	28 (2.5%)	0 (0%)
Placenta Previa, *n* (%)	0 (0%)	19 (1.7%)	1 (1.4%)
Preeclampsia, *n* (%)	52 (6.4%)	175 (16%)	3 (4.2%)
Induction of Labor, *n* (%)	111 (14%)	21 (1.9%)	5 (7.0%)
Cord Prolapse, *n* (%)	77 (9.4%)	43 (3.8%)	9 (13%)
Different Sex Twins, *n* (%)	284 (35%)	422 (37%)	28 (39%)

*Missing data: Gravidity Group n = 2013/2014

^a^n (%); Mean (SD)

Abbreviations: g, gram

**Table 2:** Fetal characteristics by mode of delivery

Variable	Vaginal, N = 1703 (42%)^*a*^	Cesarean, N = 2325 (58%)^*a*^
Female Sex, *n* (%)	871 (51%)	1139 (49%)
Gestational Age, Mean (SD)	34 (3)	34 (2)
Gestational Age Group, *n* (%)		
Moderate to Late Preterm (32 + 0 to 36 + 7)	1474 (87%)	2050 (88%)
Very Preterm (28 + 0 to 31 + 6)	98 (5.8%)	214 (9.2%)
Extremely Preterm (24 + 0 to 27 + 6)	131 (7.7%)	61 (2.6%)
Birth Weight (g), Mean (SD)	2026 (582)	2,087 (517)
Weight Group, *n* (%)		
VLBW (< 1500(g))	225 (13%)	309 (13%)
LBW (1500–2499(g))	1183 (69%)	1540 (66%)
Normal Birth Weight (= > 2500(g))	295 (17%)	476 (20%)
Low Apgar 1 Min*, *n* (%)	171 (10%)	493 (22%)
Low Apgar 5 Min*, *n* (%)	71 (4.4%)	76 (3.3%)
Breech Presentation of Either Twin (Malpresentation), *n* (%)	360 (21%)	1228 (53%)
Meconium Staining, *n* (%)	22 (1.3%)	41 (1.8%)
Fetal Distress**.** *n* (%)	41 (2.4%)	71 (3.1%)
Respiratory Disease	3.4 (11.0)	3.1 (11.2)
Neurologic Disease	7.1 (13.0)	7.4 (11.0)
Infectious Disease	1.2 (5.5)	1.3 (5.6)
Gastrointestinal Disease	4.7 (13.1)	5.1 (12.1)

^*a*^ n (%); Mean (SD); Median (IQR)

Abbreviations: *g*, gram

*Missing data: Low Apgar 1 Minute ***n*** = 3912/4028, Low Apgar 5 min 3907/4028

Table2 illustrates the background characteristics of the offspring. Offspring delivered via CD exhibited a higher prevalence of low 1-min Apgar score, while no differences were found in low 5-min Apgar score. Moderate to late and Very PTD were more prevalent in the CD group. For the extremely PTD group, VD was more dominant. Median follow-up was longer among the CD group regarding infectious diseases, while no differences were found in other morbidity follow-up times between the study groups. Mortality was reported only during the first eight days, during which 57 were recorded, 46 of which occurred on the day of birth; no significant differences in survival probability were observed between groups, as indicated by the Kaplan–Meier curve (log-rank, *p* = 0.34).

### Respiratory morbidities

**Fig. 2:**
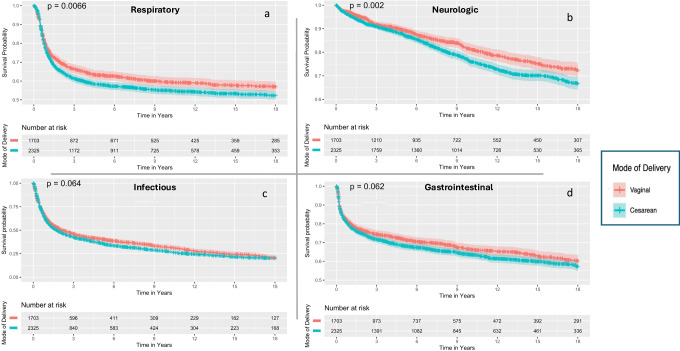
Kaplan–Meier Survival Curves by Mode of Delivery**.** Figure displays Kaplan–Meier survival curves comparing long-term health outcomes by delivery mode. Sub-figures (**a**) to (**d**) show survival probabilities for respiratory, neurologic, gastrointestinal, and infectious outcomes, with red and blue lines for vaginal and cesarean deliveries, respectively. Confidence intervals and log-rank *p*-values are included

The examination of respiratory morbidity and its subgroups (Table S2) indicated a higher incidence in the CD group, with 967 (42%) compared to 596 events (35%) in the VD group (OR 1.32, 95%CI 1.16–1.51), also evident based on the Kaplan–Meier survival curve (Fig. 2a, Log-rank *p* = 0.006). Offspring delivered via CD showing a notably higher likelihood of developing asthma (40% vs. 32%, OR 1.40, 95%CI 1.23–1.60). CD’s lasting effect was further supported by the Cox regression model, adjusted for offspring’s birth year, ethnicity, and gestational age groups (aHR 1.15, 95%CI 1.02–1.30, Table 3). A sub-analysis based on the elective-noncomplicated sub-group showed a similar trend while adjusting for the same covariates (aHR 1.14, 95%CI 1.01–1.29, Table 3). When adjusted for weight group, offspring year of birth and ethnicity, CD was not associated with long-term respiratory morbidity among any gestational age subgroup; very preterm (aHR 1.46, 95%CI, 0.97–2.21) moderate to late preterm (aHR 1.12, 95%CI 0.99–1.27), and extremely preterm group (aHR 2.89, 95%CI 0.81–10.30).

**Table 3:** Incidence, hazard ratios and adjusted hazard ratios

Entire Cohort, *n* = 4028						Elective/Non-complicated, *n* = 3282				
Disease domain	Disease Incidence (number of events, %)			Crude HR (95%CI)	aHR* (95%CI)	Disease Incidence			Crude HR (95%CI)	aHR* (95%CI)
	Vaginal^*a*^ 1703 (42%)	Cesarean^*a*^ 2325 (58%)	*P*^b^			Vaginal^a^ 1418 (43%)	Cesarean^a^ 1865 (57%)	*P*^b^		
Respiratory	596 (35%)	967 (42%)	< 0.001	1.15 (1.04, 1.28)	1.15 (1.02, 1.30)	497 (35%)	786 (42%)	< 0.001	1.16 (1.04, 1.30)	1.14 (1.01, 1.29)
Neurologic	284 (17%)	502 (22%)	< 0.001	1.26 (1.09, 1.45)	1.16 (1.02, 1.36)	229(16%)	403 (22%)	< 0.001	1.31 (1.11, 1.54)	1.20 (1.01, 1.43)
Infectious	1051 (62%)	1608 (69%)	< 0.001	1.08 (1.00, 1.16)	1.10 (1.01, 1.21)	874 (62%)	1298(70%)	< 0.001	1.08 (1.00, 1.18)	1.11 (1.01, 1.23)
Gastrointestinal	513 (30%)	810 (35%)	< 0.001	1.11 (1.00, 1.24)	1.10 (0.95, 1.27)	417 (29%)	657 (35%)	< 0.001	1.11 (1.00, 1.24)	1.15 (1.01, 1.32)

^*^Adjusted for maternal age, ethnicity, gestational age group, maternal recurrence, offspring’s birth year, gestational diabetes mellitus, preeclampsia, weight group, clustering within pregnancy

Abbrevations: *HR*, Hazard Ratio; aHR, adjusted Hazard Ratio

^a^ *n* (%) ^b^ Pearson’s Chi-squared test

### Neurologic morbidities

The analysis of neurologic morbidities and subgroups (Table S2) showed a higher incidence in the CD group, with 502 (22%) compared to 284 (17%) events in the VD group (OR 1.38, 95% CI 1.17–1.62), also supported by the Kaplan–Meier survival curve (Fig. 2b, Log-rank *p* = 0.002). A notably higher likelihood of offspring delivered via CD developing movement disorder epilepsy (OR 1.52, 95%CI 1.08–2.16), ADHD (OR 1.56, 95% CI 1.12–2.10), and degenerative and demyelination syndromes (OR 1.31, 95%CI 1.01–1.71). This observation was supported by the Cox regression model when adjusted for maternal age, offspring’s birth year, gestational age group, and ethnicity (aHR 1.16, 95%CI 1.02–1.36, Table 3). A sub-analysis based on the elective-non-complicated sub-group showed a similar trend while adjusting for the same covariates (aHR 1.20 95%CI 1.01–1.43, Table 3). When adjusted for the same covariates, CD was not associated with neurologic morbidities in the moderate to late (aHR 1.11, 95% CI 0.94–1.32) and very preterm (aHR 1.34, 95% CI 0.74–2.45) groups, while in extremely preterm, CD was found significant (aHR 4.06, 95% CI 1.18–13.9).

### Infectious morbidities

The assessment of the first episode of infection in offspring, including disease subgroups (Table S2), showed a higher prevalence of infectious events in the CD group, with 1608 (69%) versus 1051 (62%) in the VD group (OR 1.39, 95%CI 1.22–1.59), although the marginal significance, this was also supported by the Kaplan–Meier survival curve (Fig. 2c, Log-rank *p* = 0.06). A notably increased likelihood of offspring developing ophthalmic (OR 1.56, 95%CI 1.06–2.33, *p* = 0.023), respiratory (OR 1.32, 95%CI 1.16–1.51), and ears, nose and throat (ENT) infections (OR 1.50, 95%CI 1.18–1.92) compared to VD. The Cox regression model, which adjusted for maternal age, ethnicity, offspring’s birth year, and weight group, presented an association between CD and long-term infectious morbidity (aHR 1.10, 95%CI 1.01–1.21, Table 3). A sub-analysis based on the elective-non-complicated sub-group adjusted for the same covariates, showcasing CD association with infectious diseases (aHR 1.10 95%CI 1.01–1.23, Table 3). While adjusting for the same covariates, CD was found to be associated with infectious-related disease in the very preterm (aHR 1.88; 95% CI 1.30–2.71) and in the extremely preterm groups (aHR 2.55; 95% CI 1.17–5.56), but not in the moderate to late preterm group (aHR 1.04; 95% CI 0.94–1.15).

### Gastrointestinal morbidities

The evaluation of GI-related events, including subgroups (Table S2), revealed an increase in hospitalizations for GI causes in the CD group with 810 cases (35%) versus 513 (30%) in the VD group (OR 1.24, 95%CI 1.08–1.42), although the marginal significance, this was also supported by the Kaplan–Meier survival curve (Fig. 2d, Log-rank *p* = 0.06). A specifically higher likelihood of colonic functional disease in offspring delivered via CD compared to VD (OR 1.27, 95%CI 1.05–1.53). The Cox regression model adjusted to maternal age, ethnicity, IVF, and weight category did not present an association between CD and long-term GI morbidity (aHR 1.10, 95%CI 0.95–1.27, Table 3**).** A sub-analysis among the elective-non-complicated achieved significance and, while adjusting for the same covariates, with CD being associated with the outcome within this subgroup (aHR 1.15, 95%CI 1.01–1.32, Table 3). When adjusted for maternal age, ethnicity, IVF, and weight category, CD was found to be associated with GI-related disease among the very preterm (aHR 1.82, 95%CI 1.20–2.77) and the extremely preterm groups (aHR 2.60; 95% CI 1.11–6.11), while did not reach significance among the moderate to late preterm group (aHR 1.02, 95%CI 0.90–1.17).

## Discussion

### Main findings

This retrospective analysis encompassed a considerable number of preterm twin deliveries and revealed a notable increase in respiratory, neurologic, and infectious morbidities associated with CD, while GI morbidities did not reach significance.

### Interpretation

Considerable investigations have probed the relationship between delivery mode and subsequent *respiratory outcomes* in offspring, mainly focusing on the incidence of childhood asthma, yielding a range of sometimes conflicting results[10, 11, 27–31]. Within our cohort, offspring in the CD group exhibited a higher incidence of respiratory- morbidities, specifically asthma- and obstructive sleep apnea. These findings were similar in all sub-analyses. This finding aligns with a previous study on singleton term offspring, which suggests an association between term-elective CD and increased respiratory hospitalizations compared to VD [32].

Previous research on delivery mode’s impact on *neurologic outcomes*, particularly ASD, has been inconclusive [32–45]. In this cohort, ASD incidence was not affected by the mode of delivery of the offspring. The Twin Birth Study also noted no differences in neurologic outcome in twin pregnancies between planned CD and VD up to 24 months [20, 21]. This study, extending to age 18, found a higher risk of neurological morbidities in the CD group when observing the entire cohort. However, when the analysis was stratified by gestational age, no differences were noted between CD and VD in the moderate to late gestational age group at both the 2-year and 18-year follow-ups, mirroring the results of the Twin Birth Study (Supplementary Table S2; Two-year follow-up analysis). This suggests that the influence of CD on neurological outcomes might be more pronounced in younger gestational ages.

As in current findings among twins, previous research indicated an elevated long-term association between *infectious outcomes* and CD [7, 46, 47]. Recent research demonstrated increased infection hospitalizations in term-singleton deliveries born via CD as compared to VD [47], a finding supported by another study enabling a sibling-matched analysis [7]. Furthermore, studies also presented increased respiratory and GI infections in CD-born as opposed to the VD offspring [46, 48].

To the best of our understanding, minimal data is present regarding the mode of delivery’s effect on GI-related morbidities later in life. This study found increased GI incidence in offspring born via CD compared to VD. However, this effect was not evident over time. Other studies showed no significant link between CD and functional GI disorders in children aged 13–18 [49], and no difference in term singletons with breech presentation up to the age of 18 [50].

The clinical outcomes of delivery modes might be attributed to three fundamental mechanisms: neonatal microbial colonization, physiological stimuli from VD, and epigenetic changes [51]. Children born via CD may not acquire maternal microbiota diversity as effectively, potentially leading to increased risks of respiratory, GI, and infectious morbidities [52–55]. CD-associated antibiotic use may also disrupt gut microbiota, potentially heightening the risk of metabolic diseases, asthma, and IBD [56, 57]. This disrupted microbiota contributes to increased epithelial barrier permeability and inflammation, linked to various health disorders [58–60]. VD’s associated mechanical forces and the associated maturation of the hypothalamic–pituitary–adrenal axis are related to the development of the fetus's respiratory, neurologic, and immune systems [61–63]. Furthermore, delivery mode-related epigenetic modifications, particularly methylation, might affect infant health by altering gene expression [64, 65], with their long-term health implications still being investigated [66].

This study’s strengths lie in its large cohort, 18-year follow-up, and the setting of a universal healthcare system, enhancing generalizability and reducing healthcare access disparities. However, its retrospective design limits causation inference and insufficient documentation on covariates, including the number of placentas in each pregnancy, childhood exposures, and breastfeeding practices. This study exclusively involved patients from a single HMO; however, previous research suggests no significant demographic differences between the study population and those insured by other HMOs in Israel [67]. The indication for the CD, which was not reported in our database, may have affected offspring health and not the actual cesarean delivery. However, the main indications for cesarean delivery have been accounted for, and the results have remained robust. This study encompasses approximately 30 years of data, during which guidelines have changed, potentially affecting and limiting our results; to address this, we adjusted for the year of birth in our analysis. Some relevant hospital encounters may have occurred outside hospital and ambulatory services due to immigration between health maintenance organizations. Although this limitation is possible, it can be assumed that immigration rates would be similar across the different modes of delivery groups, thereby possibly mitigating the specific impact of this limitation on our findings.

## Conclusion

While CDs can be lifesaving in certain circumstances, this study highlights the potential long-term consequences for pediatric health. The observed increase in respiratory, neurological, and infectious morbidity associated with CD suggests that clinicians should evaluate the necessity of this intervention.

Considering the WHO’s concerns regarding the worldwide increase in CD rates, the complex factors influenced by this trend [68, 69], and the high rates of CD in preterm twin deliveries [2], future prospective research should study the long-term effects of delivery modes, considering factors like placental count [70, 71], breastfeeding practices [72–74], and other early life exposures [75–79]. This nuanced understanding could lead to more tailored healthcare strategies, ensuring that CD is reserved for genuine medical necessity rather than as a routine practice absent of necessity and enhancing overall maternal and child health.

## Supplementary Information

Below is the link to the electronic supplementary material.

Supplementary file1 (DOCX 1269 KB)

## Data Availability

The datasets are available from the corresponding author upon reasonable request and subject to IRB approval.

## Abbreviations

PTD: Preterm Delivery
CD: Cesarean Delivery
ASD: Autism Spectrum Disorder
VD: Vaginal Delivery
SUMC: Soroka University Medical Center
PROM: Premature Rupture of Membranes
ICD-9: International Classification of Diseases, 9th Revision
WHO: World Health Organization
IVF: In Vitro Fertilization
GDM: Gestational Diabetes Mellitus
ENT: Ears, Nose, and Throat
IBD: Inflammatory Bowel Disease
HMO: Health Maintenance Organization
RSE: Robust Standard Errors
BIC: Bayesian Information Criterion
aHR: Adjusted Hazard Ratio
SD: Standard Deviation
IQR: Interquartile Range
