# Supplementary material for: Mode of delivery among preterm twins and offspring health, a retrospective cohort study
Source: Eur J Pediatr. 2025 Mar 10;184(3):234. doi: 10.1007/s00431-025-06060-5 (PMC11893663; doi:10.1007/s00431-025-06060-5)
Supplement: Supplementary file 1 — Supplementary file1 (DOCX 1269 KB) [file 431_2025_6060_MOESM1_ESM.docx]

**Appendix**

**Figure S1**

**Figure S1** displays Kaplan-Meier curves analyzing the impact of delivery mode—vaginal or elective cesarean—on various health outcomes over time. Sub-figures (a) to (d) show survival probabilities for neurologic, respiratory, gastrointestinal, and infectious conditions. The blue line represents individuals who had elective cesarean deliveries, while the red represents non-complicated vaginal delivery, illustrated over an 18-year timeline. Shaded areas around the curves indicate confidence intervals, reflecting the precision of the survival estimates. P-values derived from log-rank tests are provided, indicating the level of statistical significance between delivery modes for each health outcome. Beneath the curves, the number of at-risk is indicated.

| **Table S1 Breech By Mode Of Delivery and Gestational Age Group** | | | | | | | | | |
| --- | --- | --- | --- | --- | --- | --- | --- | --- | --- |
| **Characteristic** | **Preterm Category: Late Preterm**, N = 3524 | | | **Preterm Category: Very Preterm**, N = 312 | | | **Preterm Category: Extremely Preterm**, N = 192 | | |
|  | **Vaginal**, N = 1,474*^1^* | **Cesarean**, N = 2,050*^1^* | **p-value***^2^* | **Vaginal**, N = 98*^1^* | **Cesarean**, N = 214*^1^* | **p-value***^2^* | **Vaginal**, N = 131*^1^* | **Cesarean**, N = 61*^1^* | **p-value***^2^* |
| **Breech Presentation** | 305 (21%) | 1,090 (53%) | <0.001 | 14 (14%) | 106 (50%) | <0.001 | 41 (31%) | 32 (52%) | 0.005 |
| *^1^* n (%) | | | | | | | | | |
| *^2^* Pearson’s Chi-squared test | | | | | | | | | |

| **Table S2**- Selected pediatric subgroup morbidities according to mode of delivery | | | | | | |
| --- | --- | --- | --- | --- | --- | --- |
| **Variable** | **Proportions** | | | **Regression Analysis** | | |
|  | **Vaginal, N = 1703 (42%)***^1^* | **Cesarean, N = 2325 (58%)***^1^* | **p-value***^2^* | **OR***^3^* | **95% CI***^3^* | **p-value** |
| 1. **Respiratory subgroup morbidities** | | | | | | |
| Asthma | 545 (32%) | 925 (40%) | <0.001 | 1.40 | 1.23, 1.60 | <0.001 |
| OSA | 26 (1.5%) | 46 (2.0%) | 0.28 | 1.30 | 0.81, 2.14 | 0.28 |
| Total respiratory events | 596 (35%) | 967 (42%) | <0.001 | 1.32 | 1.16, 1.51 | <0.001 |
| 1. **Neurologic subgroup morbidities** | | | | | | |
| Autism | 9 (0.5%) | 23 (1.0%) | 0.10 | 1.88 | 0.90, 4.30 | 0.10 |
| Myopathy | 11 (0.6%) | 9 (0.4%) | 0.24 | 0.60 | 0.24, 1.45 | 0.25 |
| Eating Disorders | 8 (0.5%) | 12 (0.5%) | 0.83 | 1.10 | 0.45, 2.81 | 0.84 |
| Sleep Disorders | 1 (<0.1%) | 9 (0.4%) | 0.05 | 6.61 | 1.24, 122 | 0.024 |
| Movement Disorder Epilepsy | 49 (2.9%) | 100 (4.3%) | 0.018 | 1.52 | 1.08, 2.16 | 0.017 |
| CP/Plegia/Palsy | 6 (0.4%) | 14 (0.6%) | 0.26 | 1.71 | 0.69, 4.85 | 0.26 |
| Psychiatric/Emotional | 53 (3.1%) | 93 (4.0%) | 0.13 | 1.30 | 0.92, 1.84 | 0.13 |
| ADHD | 71 (4.2%) | 148 (6.4%) | 0.002 | 1.56 | 1.17, 2.10 | 0.002 |
| Developmental Disorders | 52 (3.1%) | 77 (3.3%) | 0.64 | 1.09 | 0.76, 1.56 | 0.64 |
| Degenerative/Demyelisation | 91 (5.3%) | 160 (6.9%) | 0.046 | 1.31 | 1.01, 1.71 | 0.044 |
| Total neurologic  events | 284 (17%) | 502 (22%) | <0.001 | 1.38 | 1.17, 1.62 | <0.001 |
| 1. **Infectious subgroup morbidities** | | | | | | |
| Bacteremia Septicemia | 25 (1.5%) | 23 (1.0%) | 0.16 | 0.67 | 0.38, 1.19 | 0.17 |
| Invasive Bacterial Infections | 11 (0.6%) | 5 (0.2%) | 0.032 | 0.33 | 0.10, 0.91 | 0.032 |
| Neonatal Infections | 23 (1.4%) | 41 (1.8%) | 0.30 | 1.31 | 0.79, 2.23 | 0.30 |
| Ophthalmic Infections | 38 (2.2%) | 80 (3.4%) | 0.025 | 1.56 | 1.06, 2.33 | 0.023 |
| Orthopedic Infections | 7 (0.4%) | 8 (0.3%) | 0.73 | 0.84 | 0.30, 2.39 | 0.73 |
| Respiratory Infections | 995 (58%) | 1,512 (65%) | <0.001 | 1.32 | 1.16, 1.51 | <0.001 |
| Skin Infections | 89 (5.2%) | 117 (5.0%) | 0.78 | 0.96 | 0.72, 1.28 | 0.78 |
| Bacterial Infections | 36 (2.1%) | 48 (2.1%) | 0.91 | 0.98 | 0.63, 1.52 | 0.91 |
| Viral Infections | 54 (3.2%) | 124 (5.3%) | <0.001 | 1.72 | 1.25, 2.40 | <0.001 |
| Pneumonia | 86 (5.0%) | 158 (6.8%) | 0.022 | 1.37 | 1.05, 1.80 | 0.021 |
| Bronchiolitis | 140 (8.2%) | 170 (7.3%) | 0.28 | 0.88 | 0.70, 1.11 | 0.29 |
| CNS Infection | 18 (1.1%) | 28 (1.2%) | 0.66 | 1.14 | 0.63, 2.11 | 0.66 |
| ENT Infections | 107 (6.3%) | 213 (9.2%) | <0.001 | 1.50 | 1.18, 1.92 | <0.001 |
| GI Infections | 51 (3.0%) | 86 (3.7%) | 0.22 | 1.24 | 0.88, 1.78 | 0.22 |
| Total Infectious events | 1,051 (62%) | 1,608 (69%) | <0.001 | 1.39 | 1.22, 1.59 | <0.001 |
| 1. **GI subgroup morbidities** | | | | | | |
| Esophageal | 7 (0.4%) | 21 (0.9%) | 0.06 | 2.21 | 0.98, 5.62 | 0.06 |
| Anorectal | 15 (0.9%) | 26 (1.1%) | 0.45 | 1.27 | 0.68, 2.47 | 0.45 |
| Hepatitis | 4 (0.2%) | 2 (<0.1%) | 0.24 | 0.37 | 0.05, 1.88 | 0.23 |
| Surgical Obstruction/Intussusception | 2 (0.1%) | 7 (0.3%) | 0.31 | 2.57 | 0.62, 17.3 | 0.21 |
| Celiac Disease | 10 (0.6%) | 17 (0.7%) | 0.58 | 1.25 | 0.58, 2.83 | 0.58 |
| Hemorrhoids | 5 (0.3%) | 7 (0.3%) | 0.96 | 1.03 | 0.33, 3.47 | 0.97 |
| Gastroduodenal | 11 (0.6%) | 20 (0.9%) | 0.44 | 1.33 | 0.65, 2.89 | 0.44 |
| Appendix | 23 (1.4%) | 27 (1.2%) | 0.59 | 0.86 | 0.49, 1.51 | 0.59 |
| Hernia | 209 (12%) | 312 (13%) | 0.28 | 1.11 | 0.92, 1.34 | 0.28 |
| IBD | 2 (0.1%) | 2 (<0.1%) | 1.00 | 0.73 | 0.09, 6.11 | 0.76 |
| Colonic Functional Diseases | 207 (12%) | 347 (15%) | 0.012 | 1.27 | 1.05, 1.53 | 0.011 |
| Other GI | 151 (8.9%) | 227 (9.8%) | 0.33 | 1.11 | 0.90, 1.38 | 0.33 |
| Total GI events | 513 (30%) | 810 (35%) | 0.002 | 1.24 | 1.08, 1.42 | 0.002 |
| *^1^* n (%)  *^2^* Pearson’s Chi-squared test; Fisher’s exact test  *^3^* OR = Odds Ratio, CI = Confidence Interval  *Abbrevations: OSA= Obstructive Sleep Apena ,* ADHD= Attention Deficit Hyperactivity Disorder, CNS = Central Nervons System, ENT = Ear, Nose, Throat, IBD=Inflammatory bowel disease, GI = Gastrointestinal. | | | | | | |

**Two-year follow-up analysis**

| **Table S3. adjusted hazard ratios – 2 year follow up** | | |
| --- | --- | --- |
| **Disease Domaine** | **Elective/Non- Complicated**  **Adjusted* Hazard Ratio (95%CI)** | **Entire Cohort**  **Adjusted* Hazard Ratio (95%CI)** |
| Respiratory | 1.14 (1.01, 1.29) | 1.14 (1.01, 1.28) |
| Neurologic | 1.24 (1.04, 1.49) | 1.20 (1.02, 1.41) |
| Infectious | 1.11 (1.01, 1.23) | 1.10 (1.01, 1.21) |
| Gastrointestinal | 1.18 (1.03, 1.35) | 1.10 (0.97, 1.25) |
| *Adjusted for maternal age, ethnicity, gestational age group, maternal recurrence, offspring’s birth year, gestational diabetes mellitus, preeclampsia, weight group, clustering within pregnancy. | | |

**Neurological Outcomes by CD and Gestational Age Groups**

In the first two years, cesarean delivery was not associated with increased neurologic morbidity risk in moderate to late (aHR 1.17, 95% CI 0.99-1.38) and very preterm twins (aHR 1.22, 95% CI 0.67-2.23), mirroring covariate adjustments from the 18-year findings. However, in extremely preterm infants, CD significantly increased the risk (aHR 4.56, 95% CI 1.40-14.8).

| **Table S4. Mode of Delivery and Infant Order** | | | | | | |
| --- | --- | --- | --- | --- | --- | --- |
| **Characteristic** | **Vaginal**, N = 1703 | | | **Cesarean**, N = 2325 | | |
|  | **First Twin**, N = 876*^1^* | **Second Twin**, N = 827*^1^* | **p-value***^2^* | **First Twin** , N = 1,138*^1^* | **Second Twin** , N = 1,187*^1^* | **p-value***^2^* |
| **Breech Presentation** | 44 (5.0%) | 316 (38%) | <0.001 | 598 (53%) | 630 (53%) | 0.79 |
| *^1^* n (%) | | | | | | |
| *^2^* Pearson’s Chi-squared test | | | | | | |
